# Community Perceptions of Village Health Workers in Kisoro, Uganda

**DOI:** 10.5334/aogh.3325

**Published:** 2021-08-16

**Authors:** Crystal Zheng, Joanne Anthonypillai, Sam Musominali, Gloria Fung Chaw, Gerald Paccione

**Affiliations:** 1 Section of Infectious Diseases, Tulane University School of Medicine, New Orleans, Louisiana, US; 2 Department of Pediatrics, The Children’s Hospital at Montefiore, Bronx, New York, US; 3 Kisoro District Hospital, Kisoro, Uganda, UG; 4 Doctors for Global Health, Decatur, GA; 5 Albert Einstein College of Medicine, Bronx, NY, US

## Abstract

**Background:**

Village health workers (VHWs) can serve as a valuable resource to address public health needs in resource-limited settings such as Uganda. However, the successful implementation of VHW programs can be limited by poor acceptability among community members. Kisoro District Hospital (KDH) in Kisoro District, Uganda operates a VHW program and, at the time of the study, was expanding its services to 11 additional villages.

**Objective:**

The objective of this study was to evaluate community perceptions of VHWs in villages of Kisoro District with no prior exposure to the KDH VHW program in order to improve community acceptance when expanding the program to additional villages.

**Methods:**

We administered surveys to 658 community members from 11 villages to evaluate their perceptions of VHWs prior to receiving VHW services. Additionally, we conducted focus group discussions among 97 participants to explore perceptions of VHWs in further depth.

**Findings:**

Community members were generally accepting of VHWs. They preferred that VHWs provide both curative and preventive services across a broad range of health domains as opposed to a single disease. Expectations of the responsibilities of a VHW were influenced by agricultural occupational and household responsibilities, particularly for women. Participants expressed a preference to be actively involved in the selection and oversight of VHWs and that VHWs receive compensation.

**Conclusions:**

1) Community members’ expectations of VHWs are shaped by environmental, cultural, and social factors. 2) Active community engagement in the VHW program is key. 3) Aligning a VHW program with community perceptions may improve acceptance, in turn influencing effectiveness and sustainability. These findings were used to expand the KDH VHW Program into the participating villages in a manner consistent with community preferences. Our findings may provide guidance on enhancing the uptake of community-based VHW programs for VHW stakeholders and policymakers in other settings.

## Introduction

Village health workers (VHWs) are trusted members of the community with limited medical training who can provide health education, promotion, and basic healthcare services. VHW programs have been effective in multiple countries and health domains, and are an important strategy endorsed by the World Health Organization and United Nations to improve health outcomes, particularly in resource-strained areas [1,2]. However, implementation barriers continue to hinder the effectiveness of VHW programs in certain settings.

Uganda is a World Bank-classified low-income country that has attempted to implement VHW programs with limited success. The country has a critical shortage of skilled healthcare professionals, with only 0.2 physicians per 1,000 people [3]. Seventy-five percent of the population resides in rural areas, where ensuring healthcare access to those with the greatest need is even more difficult [4]. In 2001, the Ugandan Ministry of Health (MOH) created Village Health Teams (VHTs) consisting of unpaid volunteers from the community to address these public health challenges. VHTs were tasked with many responsibilities including health promotion, home visits, management of common health conditions, and disease surveillance [5]. However, the program proved to be ineffective and unsustainable, in large part due to insufficient training, poor supervision, and a reliance on volunteerism [6]. The role of existing VHTs is now mainly limited to mobilization of communities for specific health campaigns, rather than the provision of broad health services as originally envisioned [7]. To address the shortcomings of VHTs, the Ugandan MOH proposed a new Community Health Extension Workers (CHEW) program that included financial and implementation support [8]. However, the policy was withdrawn in 2019 due to a lack of resources [9].

Kisoro District is a rural region in southwest Uganda with a population of 281,700 [10]. In 2007, Kisoro District Hospital (KDH), in partnership with Doctors for Global Health and the Albert Einstein College of Medicine, established a VHW program to deliver health promotion and primary care services to surrounding communities with limited access to care. Participating villages with between 80–400 households select VHWs who are trained to provide health education [11], linkage to care [12], and treatment of common ailments across a broad range of health domains including women and children’s health, chronic disease [13], and acute illness. The KDH VHW program is separate from the national VHT program and currently includes 52 VHWs from 50 villages covering a population of roughly 40,000.

An important component of VHW programmatic success is acceptance by the community. VHWs are generally well-received. For example, studies of VHW programs in Kenya and Uganda targeting malaria, HIV, tuberculosis, and hypertension have reported that community members positively associate VHWs with providing medications, health education, linkage to the healthcare system, and bringing healthcare closer to the community [14,15]. However, in some settings, poor acceptance of VHWs due to lack of trust and a preference for the formal healthcare system has been associated with low utilization of services [16,17]. Although ultimately withdrawn, the Ugandan CHEW program outlined several goals to address sustainability, one of which was to “improve community participation, engagement, and ownership of health programmes [8].” The objective of this study was to evaluate community perceptions of VHWs in villages of Kisoro District with no prior exposure to the KDH VHW program in order to improve community acceptance when expanding the program to additional villages. The findings were used to guide future directions of the Kisoro VHW program, and may be useful for stakeholders and policymakers invested in improving the implementation of VHW programs in Uganda and elsewhere.

## Methods

The study used a mixed methods approach combining quantitative survey and qualitative focus group methods. Eleven villages in Kisoro District that had been invited to join the KDH VHW program were selected to participate in the study. Between November 2016 and January 2017, 60 households in each village were randomly selected, and one adult from each household was invited to participate. The chairperson of each village accompanied a research assistant to the households for purposes of navigation but did not stay while the survey was administered. Upon arriving at each household, the self-identified head of the household or their spouse was randomly selected by a coin flip and invited to participate in the survey. If neither were present, the oldest member of the household was invited to participate. If no adults were present, the nearest household was invited to participate.

The survey included questions about VHW responsibilities, ideal characteristics, selection process, and compensation in multiple choice or short response format (see Supplementary Data). Questions were written by the investigators with input from VHW program stakeholders and were translated and back translated from English to Rufumbira, the local language. A pilot survey was administered in two villages not included in this study and revised to create the final survey. The surveys were administered verbally on weekdays during daytime hours and lasted roughly 30 minutes. Market days were excluded to increase the likelihood of finding someone at home. Free response questions were recorded in Rufumbira and translated into English. The responses were independently coded according to similarities by two investigators, with review by the senior investigator to reach consensus. Data analysis was performed with Excel for Microsoft 365 (Seattle, WA).

Focus Group Discussions (FGDs) were performed in 9 of the 11 villages. Each village was assigned one of the following demographic groups: male, young female (age ≤ 50), and older female (age > 50), with each demographic group represented by three villages. Recruitment was by word-of-mouth with a goal of 10 participants per group. FGDs were moderated by KDH staff, conducted in community spaces, and lasted approximately two hours. Standardized questions were developed by investigators based on interim review of survey responses, and open-ended follow up questions were permitted by the moderator. In all FGDs, research assistants recorded field notes which were translated. Seven FGDs were also audio-recorded, transcribed, and translated. The translated transcriptions and field notes were independently coded for themes by two investigators, with review by the senior investigator to reach consensus.

This study was approved by the Institutional Review Board of the Albert Einstein College of Medicine (Reference # 036203) and by community and hospital healthcare leaders in Kisoro District, Uganda. Participants provided informed consent and did not receive compensation. The investigators and research assistants include individuals involved with the operation of the KDH VHW program.

## Results

A total of 658 participants from 660 randomly selected households responded to the survey. The participants were predominantly female (73%) with a median age of 34. Ninety-seven participants attended FGDs in one of three demographic groups: 24 males, 29 young females, and 42 older females (***Table 1***). Each FGD consisted of on average 11 participants (range 6–18). Responses to select survey questions are presented in ***Table 2***. Representative FGD quotes are presented in ***Table 3***.

**Table 1:** Characteristics of Study Participants.


SURVEY RESPONDENTS (n = 658)	

Female (%)	478 (73%)

Median age (interquartile range)	34 (27–46)

Median household size (interquartile range)	5 (4–7)

Households with children < age 15 (%)	509 (79%)

Average number of children per household (SD)	2.5 (1.9)

Heard of VHWs previously (%)	471 (72%)

Received VHW services previously (%)	293 (45%)

**FOCUS GROUP DISCUSSION PARTICIPANTS (n = 97)**	

Male (n = 24)	

Average age (range)	35.5 (18–66)

Married (%)	16 (67%)

Completed primary school (%)	10 (42%)

Farming occupation (%)	15 (63)

Received VHW services previously (%)	18 (25%)

**YOUNG FEMALE (n = 31)**	

Average age (range)	29 (19–50)

Married (%)	31 (100%)

Completed primary school (%)	14 (45%)

Farming occupation (%)	26 (84%)

Received VHW services previously (%)	17 (55%)

**OLDER FEMALE (n = 42)**	

Average age (range)	74 (50–101)

Married (%)	41 (100%)

Completed primary school (%)	0 (0%)

Farming occupation (%)	31 (74%)

Received VHW services previously (%)	15 (36%)


**Table 2:** Survey responses.


QUESTION (QUESTION TYPE)				RESPONSE	

*Personal characteristics and qualities of VHWs*					

What gender should a VHW be? (multiple choice) N = 655					

Male				158 (24%)	

Female				219 (33%)	

Does not matter				278 (42%)	

What is the appropriate age of a VHW? (free response)					

Median age (range)				30 (18–70)	

Is it important that a VHW have a spouse? (yes/no) N = 644					

Yes				513 (80%)	

Should a VHW be required to read and write? (yes/no) N = 647					

Yes				647 (100%)	

What are the most important qualities a VHW should possess? (free response) N = 658†*					

Educated, smart	236 (36%)		Active, mobile, energetic	93 (14%)	

Kind, caring	210 (32%)		Humble	76 (12%)	

Respectful of confidentiality, discreet	199 (30%)		Non-judgmental	53 (8%)	

Possesses good interpersonal skills, sociable	199 (30%)		Possesses good morals	42 (6%)	

Well behaved	150 (23%)		Acts like a leader or role model	37 (6%)	

Trustworthy, honest	130 (20%)		Available	31 (5%)	

Competent, hardworking, skilled	120 (18%)		Other	18 (3%)	

Clean, presentable	99 (15%)		Reserved, quiet	6 (1%)	

*Responsibilities of VHWs*					

Left: What are the most important activities a VHW should do? (free response) N = 658†*					

Right: Should a VHW perform the following activities? (yes/no) N = 647–657§¶					

Prescribe medications	605 (92%)	646 (98%)	Provide women’s health services	65 (10%)	640 (98%)

Diagnose disease	246 (37%)	628 (96%)	Promote sanitation	51 (8%)	

Provide health education	241 (37%)	652 (100%)	Promote vaccination	18 (3%)	

Perform home visits	151 (23%)		Implement public health measures	17 (3%)	

Refer to higher level of care	98 (15%)		Other	6 (1%)	

Provide goods/services	86 (13%)	633 (97%)			

*VHW selection, job characteristics, and compensation*					

Should a VHW position be open to all or be selected in some way? (multiple choice) N = 656					

Selected in some way				623 (95%)	

Who should select VHWs? (multiple choice) N = 630					

Community members				568 (90%)	

District hospital				539 (6%)	

Village/district council				21 (4%)	

Should VHWs work part-time or full time? (multiple choice) N = 655					

Part-time				377 (58%)	

Should VHWs be selected for life or for a limited period of time? (multiple choice) N = 653					

Limited period of time				512 (78%)	

Should VHWs be volunteers or paid for their work? (multiple choice) N = 650					

Paid for their work				610 (94%)	


Questions have been paraphrased for brevity. See supplement for full text. † Free response answers are grouped according to similarities. * Percentages do not sum to 100% because participants can select more than one answer. § Number of respondents to each question varies. ¶ Some free responses did not correspond to predetermined activities listed in yes/no questions.

**Table 3:** Representative focus group discussion quotes by theme.


*Role of VHWs*

Treat acute illnesses like typhoid, emergency response, check them while they’re sick, and help them with family planning, diagnosing illnesses. (Men, FN)

Diagnose and see how they are in their life, like taking measures such as BP. (Young women, FN)

Should be at the home most of the time, maybe take 1–2x per week and walk around the village. One day of the week do follow-up of people in the community, and other days people know where to find them. (Young women, FN)

Visiting community to see how they are. (Older women, FN)

Should be involved in treating them, asking them about their disease and medical treatment. (Older women, FN)

*Education level of VHWs*

Needs enough understanding to explain medications, knows what he or she is doing… cannot train someone who did not go to school. (Men, FN)

One may be educated and not available in the community whereas one who don’t have high classes but with special skills and informed may serve the community better than the highly educated. (Men)

Usually someone understands more quickly if have education. (Young women, FN)

Sometimes a woman has talent even without much schooling. (Young women)

This village has few highly educated people and so long as this person knows how to read and write. (Young women)

If they don’t know how to read and write, they can’t know how to direct people on the way of taking meds. (Older women, FN)

Zero level of education. We never went to school. (Older women)

In addition, needs to have other good qualities, not just education. (Older women, FN)

*VHW hours and payment*

Won’t be able to do all of their usual work because they will have to be around. (Men, FN)

It takes away from their [women’s] time to take care of the home, not a problem for men. (Men, FN)

We can even feel happy and the VHW will feel motivated. (Men, when asked whether the community should know about VHW compensation)

Full time because having been selected to serve the community, he will always be needed. (Young women)

Can’t specify when this person will be working because illnesses are unpredictable. (Young women)

Has to be paid [a fixed salary] cause it’s god’s will that no one got sick. (Young women, FN)

No because people would get jealous. (Older women, when asked whether the community should know about VHW compensation, FN)

The whole village should contribute to giving but only as a token of appreciation, not as a contribution to the salary but the government should pay the salary. (Older women, FN)

*Selection and accountability*

If the VHW is paid and not providing services, can report and hold them accountable. (Men, FN)

The community will meet and evaluate whether he or she is working for them or not. The hospital should supervise to make sure the services are being delivered. (Men, FN)

Yes, to give chance to others. (Young women, when asked whether VHWs should have term limits, FN)

If working well, there should be no limit. Someone should work as long as they are able to, but should be removed if not during reselection period. (Older women, FN)

*Women’s health*

Those who are enlightened accept [family planning] and those are not they don’t accept. (Men, FN)

Can go to VHW even if the opposite gender, because they are trained. (Men, FN)

Not accepted fully, many, many misconceptions abound [about family planning]. (Young women, FN)

They all said that they can approach [a male VHW] because they trust him and they know he will be secretive. (Young women, FN)

Better to have two VHWs, a male and a female. (Older women)

Male health workers who can see females is normal. (Older women, FN)

Quotes from field notes are labeled FN, otherwise quotes are directly from participants.


**Figure 1:**
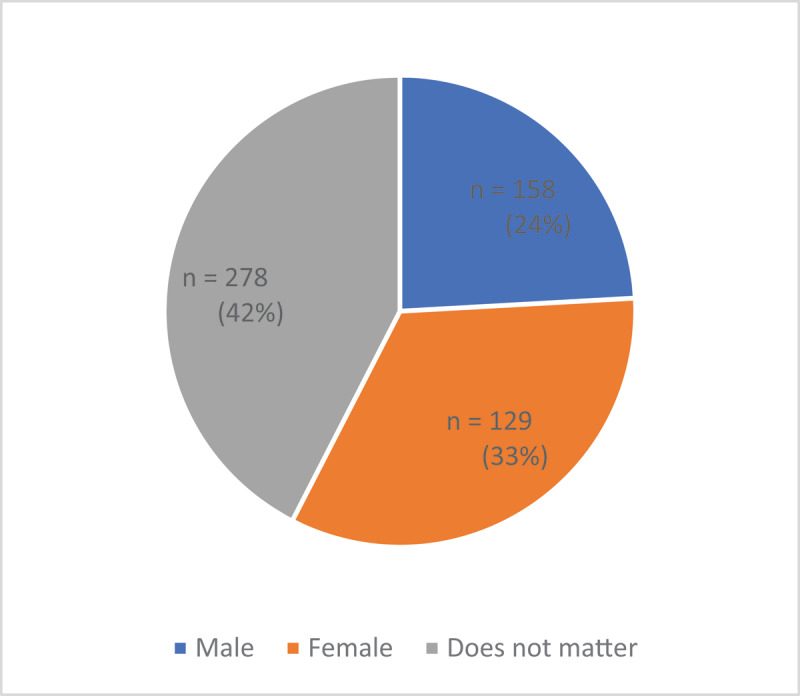
What gender should a VHW be?

Forty-two percent of survey participants indicated no preference in gender of a VHW, while 33% preferred female and 25% preferred male VHWs (***Figure 1***). Thirty-six percent of women and 32% of men preferred a VHW of the same gender. Participants who preferred female VHWs responded that women were more knowledgeable of topics affecting women and children’s health, exhibited qualities such as being kind and caring, and that women in the community would feel more comfortable being evaluated by a female VHW. Participants who preferred male VHWs responded that men were more authoritative and more available because they do not have household duties. The majority of FGD participants expressed acceptance of a male VHW providing women’s health services, citing the fact that women are frequently attended to by male healthcare workers in the hospital. Other participants stated that it could be difficult for individuals to approach a VHW of the opposite gender and suggested that each village have a male and a female VHW.

The median ideal age of a VHW reported on surveys was 30 years. Participants expressed that VHWs should be mature enough to have life experiences but still have the physical ability to be mobile throughout the community. Eighty percent agreed that a VHW should be married, explaining that married individuals are more knowledgeable about health problems faced by families, have more life experience, and are likely to remain as long-term residents of the village. Additionally, some respondents believed the VHW’s spouse could perform household duties, freeing up time for the VHW to perform his or her job duties. Many responses also described being married as a sign of moral character, or “God’s plan.”

All survey participants agreed that VHWs should at least be able to read or write Rufumbira, corresponding to an education level of primary grade 3. Most survey participants (66%) believed that completion of lower secondary school (at least S4 grade level) should be required for VHWs. In FGDs, reading and writing were considered necessary skills for VHWs in order to instruct community members in taking medications. Some believed that VHWs should be able to read and write English, the language commonly used in medical documentation and healthcare settings, in order to translate documents and present concerns of the community to the hospital. Contrary to survey results, most FGD participants believed that VHWs do not require a secondary level education and that VHW training alone is sufficient. Multiple older women believed that character or “good morals” were more important than education. One man gave a hypothetical example of someone with high education but who was unavailable in the community: “whereas one who doesn’t have high classes…may serve the community better than the highly educated.” Additionally, men, young women, and older women all expressed that a woman may be qualified to be a VHW despite not attending secondary school as long as she could read and write.

When asked in an open-ended survey question to name the most important qualities a VHW should possess, the five most frequent responses were that VHWs should be educated (36%), kind (32%), respectful of confidentiality (30%), have good interpersonal skills (30%), and be well-behaved (23%) (***Figure 3***). A specific behavior raised by 75 participants (11%) was that a VHW should “not be a drunkard” or an alcoholic. Other qualities raised were related to personality (trustworthy, humble, non-judgmental), appearance (clean, presentable), job competency (hardworking), and physical mobility (active, energetic, able to visit community members in their homes).

**Figure 2:**
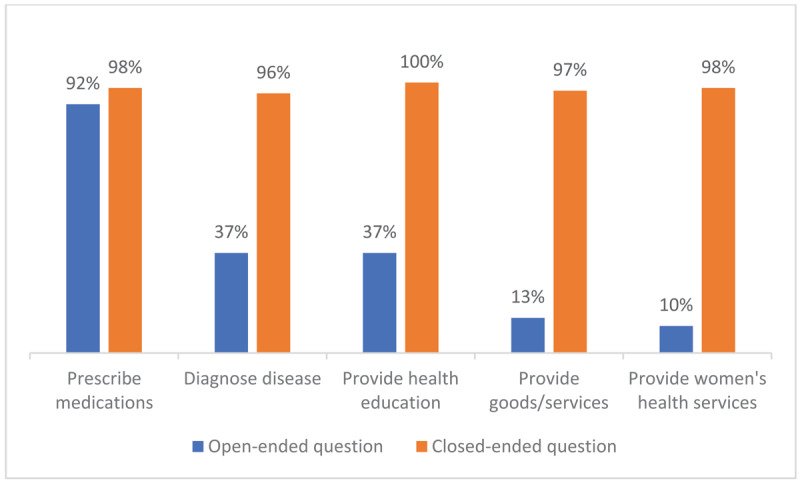
What are the most important activities a VHW should do?

When asked in an open-ended survey question to name the most important activities of a VHW, a large majority spontaneously identified prescribing medications (92%). The importance of providing medical treatment was also supported in FGDs. In contrast, the next most frequent activities, diagnosing disease and providing health education, were only volunteered by 37% of participants. Among the least frequently named activities were public health services, including promoting vaccination (3%), sanitation (8%), and women’s health services including antenatal care and family planning (10%). However, when asked about specific pre-defined services in closed-ended questions, almost all agreed that VHWs should provide health education (99.5%), discuss family planning (98%), distribute food for malnourished children (97%), and diagnose disease (96%) (***Figure 2***). Providing clothing to families (38%) and fixing a broken bicycle (17%) were not generally considered part of a VHW’s purview.

**Figure 3:**
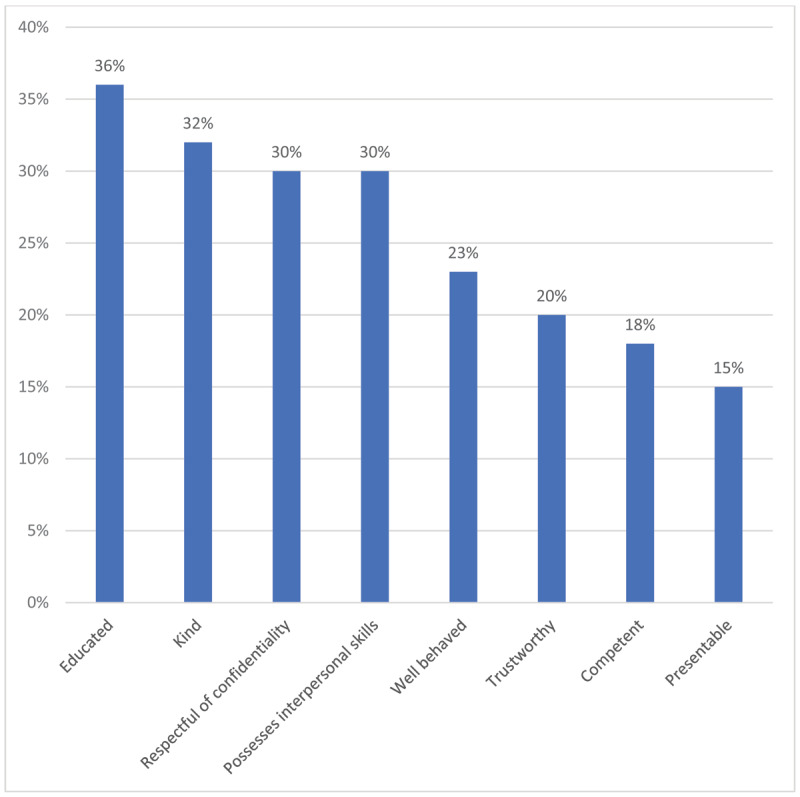
What are the most important qualities a VHW should possess?

A majority of survey participants (78%) believed VHWs should serve limited terms rather than hold lifelong positions. The median ideal term length was three years. Ninety percent believed VHWs should be selected directly by the community rather than by the district hospital or council. In FGDs from all three demographic groups, participants agreed that the community should evaluate VHW performance and that VHWs performing to the standards of the community should be able to serve for longer terms if reselected. Some participants also suggested that the VHW program hold village meetings on a regular basis to elicit community feedback.

A majority of survey participants believed VHWs should work part-time (58%) as opposed to full-time. Those who answered part-time responded that a VHW should work a median of eight days per month (IQR 4–12) and a median of six hours per day (IQR 5–8). Farming and owning a business in the village were considered ideal full-time occupations for VHWs because of their flexible schedule and presence in the community. Teaching, government work, and owning other businesses outside of the village were considered difficult occupations for VHW work.

Most survey respondents believed VHWs should be paid for their work (94%) as opposed to acting as volunteers. When asked how VHWs should be paid, 75% percent (n = 461) selected a fixed amount as opposed to fee-for-service. A majority believed villagers should contribute to VHW pay (n = 490, 77%). FGDs further supported the preference for a fixed payment rather than fee-for-service model. Both men and young women believed that a VHW should be paid for days not actively performing VHW duties because the VHW still has to be available at all times for unexpected health issues that arise. Furthermore, some participants compared a VHW position to government or hospital jobs that receive a fixed salary. When asked whether the community should contribute towards VHW compensation, many participants agreed but were divided on the mechanism. Some stated that everyone in the community should make a monthly contribution, while others stated that only those who receive services directly should pay. All groups stressed that any contribution from the community should be given as a “token of appreciation” to supplement a fixed salary paid by the government or the sponsoring organization. Groups were also divided on whether the community should know about VHW compensation. Participants expressed that they would feel “happy” knowing that the VHW was paid, be more comfortable seeking services, and be able to hold the VHW accountable. Others stated that payment was a private matter between the employer and employee and that “people would get jealous.”

## Discussion

In this study, we used a mixed methods approach to evaluate community perceptions of VHW characteristics, responsibilities, and program implementation in Kisoro District, Uganda. Similar to previously published studies of VHW programs in other settings, we found that community members generally had positive perceptions of VHWs and the services they provide.

### Ideal VHW Characteristics

A plurality of survey participants reported no preference regarding the gender of a VHW. In addition, FGD participants of both genders expressed the opinion that male VHWs can attend to women. These results are contrary to other studies from Tanzania, Somalia, and Nigeria that have reported a preference by women to receive services from female VHWs, particularly in the realm of reproductive health [18,19]. Our results may be limited by social-desirability bias due to the influence of male and hospital-affiliated interviewers, and further study on gender preference in this population is needed.

Our data on community preferences for VHW education is conflicting. Most survey respondents thought that VHWs should have a secondary level education, though this belief was not supported upon more detailed discussion in FGDs. Beyond the ability to read and write, FGD participants expected VHWs to receive targeted training for their specific activities. In Uganda, women are often not afforded the same educational opportunities as men. For example, among KDH VHWs, 100% of men but only 44% of women had at least some secondary education. A requirement for a specific level of schooling would disproportionately exclude women and be particularly limiting in communities with a preference for female VHWs. Rather than implementing education requirements, VHW programs should invest resources into developing a robust training curriculum in order to build community trust.

A number of important qualities a VHW should possess are worth highlighting. Many participants expressed that a VHW should be respectful of confidentiality. In addition to the usual privacy concerns regarding health, confidentiality may be especially important to villagers in a tight-knit community where they are likely to know one another and the VHW. Physical mobility was valued because the terrain in remote villages is difficult and a VHW would need to travel long distances on foot in order to reach each household. Older women in particular, a group that is more likely to have decreased mobility, expressed a desire for VHWs to visit them in their homes, and by extension, have the physical ability to do so. Moral character was also emphasized by older women and was mentioned in relation to marriage and being well-behaved. With respect to moral behavior, many respondents were specifically concerned about alcohol, reflective of the high prevalence of alcohol abuse (10%) among Ugandan men [20].

### Responsibilities

Dispensing medications and diagnosing disease were the most frequently identified roles of a VHW when asked in an open-ended way. Patterns of preference for VHWs to perform curative rather than preventive services have been demonstrated elsewhere in Uganda and Nepal.[20,22] However, when prompted with closed-ended questions, almost all participants agreed that VHWs should perform preventive health services, including health education and family planning. The discrepancy between the open- and closed-ended responses suggests that participants state their preferences in line with their personal experiences of the current healthcare system rather than their true preferences. In Uganda, receiving a prescription for medications is a central part of one’s expectation of a healthcare encounter [21]. Nevertheless, when presented with the option, participants expressed a preference for preventive health services as well. Additionally, participants preferred VHWs to engage in activities that can be applied across a range of health topics rather than to specific diseases. VHW programs that adopt a ‘horizontal’ rather than a “vertical” approach may be able to better serve communities that have multiple health needs but limited access to healthcare.

### Implementation

The expectations of VHW hours were informed by the farming and household responsibilities typical for an individual living in rural Uganda. Traditional gender roles impact women, in particular, who bear most of the household responsibility. Most participants expected VHWs to work part-time on certain days of the week, but to have an on-call role in which they are accessible at all hours even while continuing their principal occupation.

Many participants preferred to have an active role in the VHW program, including electing and holding the VHW accountable. Election of VHWs by the community members themselves is a mechanism to increase acceptability, since VHWs would be inherently selected based on community preferences. The enforcement of term limits was one method to ensure accountability, as reelection would depend on performance. However, the preference for term limits varied widely, and the shorter durations (as short as one year) would not be feasible or cost-effective for comprehensive, horizontal programs with extended training requirements.

Contrary to some concerns that financial incentives can undermine relationships with the community [22], most participants believed VHWs should be paid in order to incentivize service utilization and accountability. Many participants were even willing to contribute to VHW pay. While KDH uses a fee-for-service model, most participants believed that VHWs should receive a fixed payment. However, this may not reflect a true preference between the two payment models. Rather, participants may be referencing how providers are paid in the formal healthcare system with which they are familiar.

### Alignment of the Kisoro VHW Program with study findings

This study took place in 11 villages prior to the expansion of the KDH VHW program into those villages. We found that the KDH VHW program in villages with existing VHWs was aligned with many of the reported community perceptions from this study and was therefore likely to be accepted by the 11 target villages.

KDH VHWs are mostly farmers who perform health activities two days a week and can be easily located working in the fields adjacent to their homes at other times for emergencies. They are selected by an election among community members, receive financial stipends, and up to now have not had enforced term limits. Interestingly, early VHW cohorts strongly objected to both the imposition of term limits and village-wide awareness of their stipends, feeling that such knowledge would erode the community’s trust in them. Indeed, determining whether community members agreed with VHWs on these points was a strong motivation for the study.

Financial compensation in Kisoro is provided on a performance-based incentives (PBI) model, similar to fee-for-service, in which VHWs receive remuneration for completing tasks or achieving certain targets. Although the majority of participants indicated a preference for fixed payments, in a previous study we found that PBI incentivized health services delivery and improved accountability, which ultimately may improve acceptability by the community [23,24]. Regardless of the mechanism, the communities in this study strongly believed that VHWs should be remunerated, and the fact that they are remunerated, if not the particulars, should be made known.

KDH requires its VHWs to be able to read and write Rufumbira but does not require a specific level of education. VHW candidates must complete an initial 90-day training program over 18 months before serving in the community. Thereafter, active VHWs participate in two-day refresher trainings quarterly and half-day on-site supervision twice a month. In other villages we have found that such rigorous training increases the trust of the VHW in the eyes of the community, corroborating the FGD participants who expressed a preference for strong VHW-specific training over general education requirements [28].

KDH VHWs perform both curative and preventive activities across multiple health domains. VHWs are given a doctor bag filled with items such as first aid supplies, oral rehydration solution, acetaminophen, and amoxicillin and trained to apply algorithms to treat simple symptoms or refer to a higher level of care. A well-established chronic disease program allows VHWs to manage disease and provide medications for community members who would otherwise have infrequent follow-up due to the often-considerable distance to the referral hospital. VHWs also deliver “home talks,” promote immunization, family planning, and disease screening, and identify cases in their communities for public health purposes. The broad range of health services provided by the KDH VHW program aligns with the VHW activity expectations expressed by the study participants.

### Strengths and limitations

Existing studies on community perceptions have been retrospective, evaluating the perceptions of individuals living in communities already receiving VHW services. Results of these studies may be biased by exposure to the program and by a desire to provide responses that are acceptable to the interviewer, who is generally from the VHW program themselves. To our knowledge, this study is unique in that it is the first to evaluate community perceptions in a population prior to the implementation of a VHW program. Compared to existing studies relying mostly on focus groups with small sample sizes, this study used a mixed-methods design and included a large sample size from a difficult to reach population. The study took place in 11 of the most remote villages of Kisoro District, which are the villages most in need of VHW services due to their distance from the formal healthcare center.

The study had several limitations. Participants were predominantly young women because they were more likely to be home compared to men and more likely to identify as the head of the household compared to older women. Additionally, as discussed previously, participants may be referencing familiar institutions such as the government and formal healthcare sectors rather than reporting their true preferences. Although the participants did not have pre-existing exposure to the KDH program, some had exposure to the national VHT program which may have influenced their perceptions of VHWs.

## Conclusion

An effective VHW program could provide a solution to meet Uganda’s public health needs given its shortage of health professionals. However, several previous attempts at implementing VHW programs in the country have been unsuccessful. While VHW programs are generally well-accepted, challenges with effectiveness and sustainability in certain settings have been attributed to poor acceptability by the community. This study used a mixed methods approach to evaluate community perceptions of VHWs in Kisoro District, Uganda. Several conclusions are suggested by our findings: 1) Community members’ expectations of VHWs are shaped by environmental, cultural, and social factors, 2) Active community engagement in VHW selection and oversight is key, 3) Aligning a VHW program with community perceptions may improve acceptance, in turn influencing effectiveness and sustainability. The study informed the expansion of the KDH VHW program into the villages included in this study in a manner acceptable to their communities. These findings may benefit programs in Uganda and other low and middle-income countries where VHWs could fill a critical need but have been limited by poor community acceptability.

## Additional File

The additional file for this article can be found as follows:

10.5334/aogh.3325.s1 Supplementary Data. Survey instrument.
