# Supplementary material for: Community Perceptions of Village Health Workers in Kisoro, Uganda
Source: Ann Glob Health. 2021 Aug 16;87(1):82. doi: 10.5334/aogh.3325 (PMC8396136; doi:10.5334/aogh.3325)
Supplement: Supplementary Data. — Survey instrument. [file agh-87-1-3325-s1.pdf]

Supplementary Data: Survey instrument

Subject Gender ________ Age ________ Role in Household____________# Members in Household_______ # Children < 15 __________

1) **Mbese wari wunvaho kubasaho bo mu byaro?** **Yego**/Yes **Oya**/No
Have you ever heard of VHWs (or CHWs or VHTs) previously?
   a) **Mbese wari wakoresha ho ubuhereza bw'umusaho wo mu caro?** Yego Oya
   Have you ever used VHW (or CHW or VHT) services?
      i) **Niba ari yego, nubuhe buhereza batanga?**
      If yes, what services did they provide?
      ____

2) **Mugutekyereza kwawe ninkibihe bintu 2 nangwa 3 byingenzi ukora nkuwibyubuzima mucyaro agomba gukora? (*Uwubaza abaye ashubije bimwe mubisubizo biri hepfo zanditswe mukibazo gikurikiyeho, ubyandike kandi ushire akazungurize kuri yego*.)** In your opinion, what are the 2 - 3 most important activities or services a VHW should do? *(If only 1-2 responses, prompt again) [If respondent gives any of the answers suggested in the next question, fill in and also circle Yes below)*

3) **Hari ibintu bimwe abakozi bibyubuzima mu byaro bagomba gukora nibyo batagomba gukora. (*ruhuka*) Ngiye kuvuga ibintu bimwe, kandi nkubaze niba utekereza bagomba kubikora cyangwa kutabikora.** There are some things that health workers SHOULD DO and other things that health workers SHOULD NOT DO. *(pause)I* am going to name some activities, and ask you whether you think a VHW should or should not do them: Should they…
- **Gusuzuma uburwayi?** diagnose disease? Yego/ Oya
- **Kwandika amadagara?** prescribe medications? Yego/ Oya
- **Guha imiryango ibyambara?** provide clothing to families? Yego/ Oya
- **Kwigisha abaturagye ibyubuzima n'uburwayi?** Educate community members about health and disease? Yego/ Oya
- **Guha ibyokurya abana barwaye indwara zimirire mibi?** provide food for malnourished children? Yego/ Oya
- **Kuganira kukubarira urubyaro?** discuss family planning? Yego/ Oya
- **Gukanika igare yononekaye?** help fix a broken bicycle? Yego/ Oya
- **Gukebera abagore batwite?** provide antenatal care? Yego/ Oya

   a) ***Niba ari oya*; kuki washubije oya kuri….(andika buri kimwe)** *If No, ask:* Why did you respond "No" to… (fill in each one)
   Kuki wavuze oya ku Why did you respond "No" to _________________________________:
   Kuki wavuze oya ku Why did you respond "No" to _________________________________:
   Kuki wavuze oya ku Why did you respond "No" to _________________________________:

   b) ***Babaye bashubije Yego kubibazo byose, baza,*** **Mbese harico abasaho bicaro batagomba gukora?**
   If they say yes to all questions, ask: Is there anything a VHW should NOT do?

4) **Turikugerageza kumenya imyifatire nimitwarize yuwo ukora nkuwibyubuzima mucaro agomba kuba afite. (*Urekyezemo*). Ninkizihe ngeso nziza 2 -3 zingenzi utekyereza zo umukozi wibyubuzima mu caro agomba kuba afite? *Abaye ashubije 1- 2 ongera ubaze.*** We are trying to learn more about qualities and characteristics that you feel are important in a VHW. (*Pause.*) What are the 2-3 most important qualities or characteristics you think a VHW should possess? *If only 1-2 responses, prompt again.*

   a) **Mungeso itatu zivunzwe haruguru, niyihe utekereza <u>ko ari iyingenzi cane</u>? *(Zinguriza nkungeso ivuzwe ko ari iyingenzi)*** Of the **3 qualities that you named above**, which do you consider the **<u>most important</u>**? *(<u>Circle</u> the most important)*

5) **Dushaka kumenya igitsina cumuntu ushaka ngo abe umusaho wibyubuzima mu caro**
We are interested in what type of person you think a VHW should be:

   a) **Agomba kuba ikihe <u>gitsina</u> ?** What <u>gender</u> should a VHW be?
   [ ] **Igitsina gabo**/Male [ ]**Igitsina gore**/ Female [ ] **Uwariwe wese**/ It does not matter
   Kuki? Why?

   b) **Niyihe <u>myaka</u> ishitse yumusaho wibyubuzima mu caro?** What is the appropriate <u>age</u> of a VHW?
   Kuki /Why?

c) **Mbese nibyiza ko VHW <u>agira umufasha</u>, umugore cyangwa umugabo?**
Is it important that a VHW <u>have a spouse</u>, a husband or wife? Yego Oya
Kuki ? Why?

d) **Mbesi umukozi wibyubuzima mu caro akwiye kuba harurugero <u>rwamashuri</u> yarangije?**
Should a VHW be required to have a certain level of <u>education</u>? Yego
Oya

Ashubije Yego, mubaze : **uruhe rugero** *? If respondent says Yes, ask*: what grade?

e) **VHW agomba kwifuza <u>kugusoma no kwandika</u>?** Should a VHW be required to <u>read and write</u>? Yego Oya

Dushaka kumenya uko bagomba kutoranywa. We are interested in knowing how a VHW should be chosen.

6) **Mbese buri <u>muntu wese ushaka</u> kuba umusaho wicaro agomba kububa, <u>nangwa agomba gutoranywa</u>?**
Should <u>*anyone* interested</u> in being a VHW be allowed to become one, or should a VHW be <u>selected in some way</u>?

[ ]**Buri muntu ushaka**/Anyone interested [ ] **Gutoranywa**/Selected

7) **Mbese icaro kigize VHW 1-2, <u>ninde ugomba kubatoranya.</u>** ***Soma buri kimwe kiri hepfo***
If a village could have only 1-2 VHWs, <u>who should select them</u>? *Read all of the following options:*

[ ]**Agakiko ko ku caro**/ village council [ ]**Ivuriro rya Kisoro**/ Kisoro District Hospital?
[ ] **Agakiko ko ku** District/district council? [**]** **Abaturage bose munama yumugi**/all community members at a town meeting?
[ ]**Abandi**/ Other ______________________________

8) **Utekereza VHW agomba gutanga igihe kingana iki co kuba umusaho wicaro**? How much time do you think a VHW should devote to being a VHW?

a) **Utekereza VHW agomba gukora umunsi wose buri munsi atekugira undi murimo, cangwa akore iminsi mike nka vhw agire undi mirimo?** Do you think a VHW should work all day every day (full-time) and not have another job, or part-time as a VHW and have another job?

[ ] **Umunsi wose buri munsi(igihe cose)**/all day every day (full-time) [ ] **Iminsi imwe**/part-time

ABAYE YASHUBIJE GUKORA IMINSI IMWE HARUGURU,IF ANSWERED PART-TIME ABOVE,

i) **Abaye akora iminsi imwe agakora undi murimo, <u>niyihe indi mirimo</u> <u>yashoboza neza</u> VHW gukora undi murimo wibyubuzima mu caro.** If part time and working another job, <u>which other jobs</u> would make the VHW <u>more flexible</u> to do health related work in the community as a VHW?

(1) **Mbese byashoboka bite ngo VHW ashobore gukora umurimo wibyubuzima?**
How would they make it more flexible for a VHW to do health related work?

ii) **Abaye akora iminsi agakora undi murimo, <u>niyihe iyindi mirimo</u> <u>yamunaniza</u> gukora imirimo yubuvuzi mu caro**
If part time and working another job, <u>which other jobs</u> might make it <u>more difficult</u> to do health related work?

(1) **Ni kuki yamunanizagukora imirimo yibyubuzima?** Why would they make it difficult to do health related work?

iii) **Abaye akora iminsi imwe, yakora <u>iminsi ingahe buri kwezi</u> nkumusaho wicaro**.
If part-time, how many <u>days per month</u> should a part-time VHW work as a VHW?

iv) **Abaye akora iminsi imwe, yakora <u>amasaha angahe mumunsi</u>**
If part-time, how many <u>hours per day</u> should a part-time VHW work as a VHW?

b) **Mbese ba VHW bagomba gutanga ubuhereza kugeza giheki-ubuzima bwabo bwose (ntakongera gutoranywa), nangwa kumara igihe (bakongera bagatoranya)?** How long should a VHW serve their community - for life (without re-selection) or for a period of time (with re-selection)?

[ ] **Ubuzima bwose, ntakongera gutoranywa/** For life, without re-selection
[ ] **Kugira igihembwe, bakongera bagatoranya/** For a period of time, with re-selection

i) **Niba ari kumara igihe, <u>bagomba</u> gutoranywa giheki?** If for a period of time, <u>how often</u> should they be re-selected?

[ ] **Buri myaka itatu**/Every 3 years
[ ] **Buri myaka 5**/Every 5 years
[ ]**Buri myaka 7**/ Every 7 years
[ ] **Buri myaka 10**/Every 10 years

9) **Mbese VHW agomba kuba inkorera bushake, akanga gushashurwa, nangwa baje bahemberwa imirimo yabo?** Should a VHW be a volunteer and accept NO payment or should VHWs be paid for their work in some way?

[ ] **Inkorera bushake, nta mushara**/Volunteer, NO pay
[ ] **Gushashurwa**/Paid

Bashubije inkorera bushake, simbuka uje ku namba ya 10, bashubije gushashurwa, kumeza ku (a) na (b)
If they answer volunteer, skip to number 10. If they answer "paid", continue to (a) and (b).

a) **Babaye bashashuwe, bashashurwe bate/: buri buhereza batanze, nangwa umushara uhamye udashingiye kumusaha bakora** IF PAID, how should they be paid: per service they provide or a fixed salary regardless of # of hours worked?

[ ] **Buri buhereza batanze**
Per service they provide

[ ] **Umushara uhamye udashingiye kumasaha bakora**
Fixed salary, regardless of # of hours worked

b) ***BABAYE BASHASHUWE*, nubuhe buhereza bagomba gutanga *?* *IF PAID***, which services should they be paid for?

- **Gusuzuma uburwayi?** diagnose disease? Yego/ Oy
- **Kwandika amadagara?** prescribe medications? Yego/ Oya
- **Guha imiryango ibyambara?** provide clothing to families? Yego/Oya
- **Kwigisha abaturagye ibyubuzima n'uburwayi?** Educate community members about health and disease? Yego/Oya
- **Guha ibyokurya abana barwaye indwara zimirire mibi?** provide food for malnourished children? Yego/Oya
- **Kuganira kukubarira urubyaro ?** discuss family planning? Yego/Oya
- **Gukanika igare yononekaye?** help fix a broken bicycle? Yego/Oya
- **Gukebera abagore batwite ?** provide antenatal care? Yego/Oya

10) **Mbese bashshuwe agashara gake, abantu bagomba guteranya ho?** Yego/Oya
If the VHWs are paid a small amount, should people in the community be contributing to their payment
